# Neighborhood Deprivation, Indoor Chemical Concentrations, and Spatial Risk for Childhood Leukemia

**DOI:** 10.3390/ijerph20043582

**Published:** 2023-02-17

**Authors:** David C. Wheeler, Joseph Boyle, Matt Carli, Mary H. Ward, Catherine Metayer

**Affiliations:** 1 Department of Biostatistics, School of Medicine, Virginia Commonwealth University, One Capitol Square, 830 East Main Street, Richmond, VA 23298, USA; 2 Occupational and Environmental Epidemiology Branch, Division of Cancer Epidemiology and Genetics, National Cancer Institute, Rockville, MD 20850, USA; 3 School of Public Health, University of California Berkeley, Berkeley, CA 94704, USA

**Keywords:** childhood leukemia, neighborhood deprivation, Bayesian index model, selection bias

## Abstract

Leukemia is the most common childhood cancer in industrialized countries, and the increasing incidence trends in the US suggest that environmental exposures play a role in its etiology. Neighborhood socioeconomic status (SES) has been found to be associated with many health outcomes, including childhood leukemia. In this paper, we used a Bayesian index model approach to estimate a neighborhood deprivation index (NDI) in the analysis of childhood leukemia in a population-based case-control study (diagnosed 1999 to 2006) in northern and central California, with direct indoor measurements of many chemicals for 277 cases and 306 controls <8 years of age. We considered spatial random effects in the Bayesian index model approach to identify any areas of significantly elevated risk not explained by neighborhood deprivation or individual covariates, and assessed if groups of indoor chemicals would explain any elevated spatial risk areas. Due to not all eligible cases and controls participating in the study, we conducted a simulation study to add non-participants to evaluate the impact of potential selection bias when estimating NDI effects and spatial risk. The results in the crude model showed an odds ratio (OR) of 1.06 and 95% credible interval (CI) of (0.98, 1.15) for a one unit increase in the NDI, but the association became slightly inverse when adjusting for individual level covariates in the observed data (OR = 0.97 and 95% CI: 0.87, 1.07), as well as when using simulated data (average OR = 0.98 and 95% CI: 0.91, 1.05). We found a significant spatial risk of childhood leukemia after adjusting for NDI and individual-level covariates in two counties, but the area of elevated risk was partly explained by selection bias in simulation studies that included more participating controls in areas of lower SES. The area of elevated risk was explained when including chemicals measured inside the home, and insecticides and herbicides had greater effects for the risk area than the overall study. In summary, the consideration of exposures and variables at different levels from multiple sources, as well as potential selection bias, are important for explaining the observed spatial areas of elevated risk and effect estimates.

## 1. Introduction

Leukemia is the most common childhood cancer, and its etiology is likely multifactorial [1,2]. Acute lymphocytic leukemia (ALL) accounts for about 80% of childhood leukemias in most Western countries [3,4,5]. Incidence peaks at 2–5 years of age, indicating that early-life exposures are important. The incidence of ALL is highest in industrialized countries [2], and incidence rose significantly over the period 1975–2004 in the United States, Japan, and Europe [3,4,5,6]. More recent papers also show increases in childhood leukemia in some areas [7,8,9,10]. The incidence trends suggest that environmental exposures or lifestyle changes play a role in the etiology of childhood leukemia. Epidemiologic studies have implicated residential and parental exposure to chemicals such as pesticides, paints, solvents, and air pollution as risk factors for childhood leukemia [1,2]. Most studies have relied primarily on self-reports and do not identify specific compounds [1,2].

In contrast, the California Childhood Leukemia Study (CCLS) evaluated residential exposure to specific persistent chemicals measured in house dust and found significant positive associations between the concentration of individual and/or total polychlorinated biphenyls (PCBs) [11], polybrominated biphenyl ether (PBDEs) flame retardants [12], specific herbicides [13], and polycyclic aromatic hydrocarbons (PAHs) [14], and childhood leukemia risk. In our previous CCLS analyses, we also assessed simultaneous exposures to six or seven groups of chemicals measured in house dust using Bayesian group index models [15,16] and found a significant positive association for PAHs and childhood leukemia overall [15], as well as for herbicides among children who were born and raised in the home where the dust samples were taken [16]. Many of the chemicals in the CCLS data, especially congeners within chemical groups, are strongly correlated (r > 0.6), and thus, they cannot be analyzed together with traditional regression methods. Bayesian group index regression is well-suited for mixture analyses of groups of correlated exposures. The variables included in our analysis with Bayesian grouped index models were all measured within the household or determined for the individual child. These include the chemical concentrations in the different indices and the covariates such as age, ethnicity, parents’ education, and household income.

However, there could be additional risk factors from the environment outside the home. For example, geographic differences in socioeconomic status (SES) could be related to the risk of childhood leukemia. Neighborhood SES has been studied for many health outcomes, including childhood leukemia [17,18,19,20,21,22]. Area deprivation was not found to be associated with acute lymphoblastic leukemia (ALL) [22], and childhood leukemia was also not associated with area SES in some studies [19,20]. However, lowest area SES was found to be associated with lower ALL risk compared with highest area SES [21], and childhood leukemia incidence in England and Wales was higher in relatively affluent communities [18]. Using a neighborhood SES or deprivation index estimation approach, we previously found neighborhood SES to be significantly and positively associated with colorectal cancer screening adherence [23]. Neighborhood deprivation was found to be significantly and positively associated with elevated blood lead levels in children [24] and with prenatal smoke exposure [25]. An association between area SES or deprivation and childhood leukemia could exist beyond the household level in this study. In addition, unmeasured environmental factors beyond SES could lead to an increased risk for childhood leukemia.

In this paper, we used a Bayesian index model approach to estimate a neighborhood deprivation index (NDI) and its association with childhood leukemia in the CCLS. We also considered spatial random effects in the Bayesian index modeling approach to identify any areas of significantly elevated childhood leukemia risk not explained by neighborhood deprivation or individual covariates, as well as indoor chemical concentrations. Given the differential participation by SES, especially among controls, we also conducted, for the first time, simulation studies for both the NDI and spatial risk effects to address possible selection bias.

## 2. Materials and Methods

### 2.1. Study Data

The CCLS is a population-based case-control study in 35 counties in California, including 17 counties in the San Francisco Bay area and 18 in the Central Valley [11,26]. Between 1995 and 2012, cases ≤14 years old were ascertained within 72 h of diagnosis from nine major pediatric clinical centers in the study area. Using California birth certificate information, controls were matched to cases on the basis of date of birth, sex, Hispanic ethnicity, and maternal race. Parents of both case and control participants were initially interviewed to gather information about their child’s exposure to suspected leukemia risk factors. Families who had not moved since the child’s diagnosis date (reference date for controls) were eligible for a second interview (Tier 2), during which carpet dust samples were collected and information about pesticide use was obtained. The second interview and dust sampling were limited to cases and controls <8 years old (diagnosed December 1999 to June 2006) to ensure the samples reflected early-life chemical exposure of the child. Case-control matching was not maintained due to residential eligibility criteria and voluntary participation. There were 731 participants for the Tier 2 interviews (324 cases and 407 controls). Of these, 296 cases (91%) and 333 controls (82%) agreed to participate. Due to insufficient dust or interferences in the chemical analyses, some samples were not quantified, leading to a final 277 cases and 306 controls (n = 583) [13].

The dust samples were collected using either a high-volume small surface sampler (HVS3) or a household vacuum cleaner. As described previously [26], concentrations of 64 organic chemicals (ng/g dust) were measured using gas chromatography/mass spectrometry (GC/MS) in multiple ion-monitoring mode after extraction with three different extraction methods. Nine metals were measured using microwave-assisted acid digestion combined with inductively coupled plasma/mass spectrometry (ICP/MS).

Our analysis investigated the association of 67 chemicals with the risk of childhood leukemia. Out of the entire CCLS dataset, only chemical exposure variables with at least 20% non-missing observations were included, as past experience has shown that higher levels of missingness contribute negligible information on potential relations with an outcome. We organized exposures into seven chemical class indices: polychlorinated biphenyls (PCBs), polycyclic aromatic hydrocarbons (PAHs), insecticides, herbicides, metals, tobacco exposure markers of nicotine and cotinine, and polybrominated diphenyl ethers (PBDEs). The reason for these groupings was that the chemicals share a structural similarity (e.g., PCBs, PAHs, metals) or usage (e.g., herbicides, insecticides). Most missing data were the result of findings below a chemical’s detection limit. Additionally, there were missing data in the PBDE chemicals due to insufficient levels of house dust for analysis. Both types of missing observations were imputed with log-normal distributions, as previously described [15].

The interviewers took a global positioning system reading of the residence coordinates, which were linked to census data for the computation of the neighborhood deprivation index and used to evaluate spatial random effects for unexplained spatial heterogeneity in leukemia risk.

### 2.2. Neighborhood Data

We used American Community Survey (ACS) 2005–2009 five-year estimates for neighborhood variables at the census block group level related to housing, income, poverty, education, and race/ethnicity. Specifically, the variables used in the deprivation index were Black segregation, Hispanic segregation, percent of households with a ratio of income to poverty <1 (below the poverty threshold), percent receiving public assistance income, percent of renters, percent with no high school diploma, percent in a different house than 1 year ago, percent foreign born, per capita income, median house value, and median gross rent. We reversed the order of per capita income, median house value, and median gross rent to achieve a direction of association similar to the other variables and increasing with increased deprivation. We applied ACS data for the census block groups containing the residence of each study participant.

### 2.3. Statistical Models

We first used a Bayesian index model to estimate a neighborhood deprivation index and its association with childhood leukemia. We modeled the probability of childhood leukemia as

(1) $$\mathrm{logit}\left(p_{i}\right)=\beta_{0}+\beta_{1}\left(\sum_{j=1}^{C_{1}}w_{j1}q_{ij1}\right),$$

where $\beta_{1}$ is the neighborhood deprivation effect for the index with $C_{l}=11$ of SES-related neighborhood variables, $w_{j1}$ is the weight for the *j*th variable in the index, $q_{ij1}$ is the quantile for the *j*th variable in the index for the *i*th subject. We used quantiles to account for different scaling of the variables and used deciles as the quantiles (ten equally-sized groups). This approach reveals the relative importance of each area variable used in the index for childhood leukemia.

We next adjusted for the individual covariates of child age, child sex, child race/ethnicity, household income, mother’s education, mother’s age, and an indicator if residence of sampling was the residence since birth. The model was

(2) $$\mathrm{logit}\left(p_{i}\right)=\beta_{0}+\beta_{1}\left(\sum_{j=1}^{C_{1}}w_{j1}q_{ij1}\right)+z_{i}^{T}\phi ,$$

where $z_{i}$ is the vector of covariates for the *i*th subject with associated vector of effects ϕ. The race/ethnicity variables were Hispanic, non-Hispanic other, and non-Hispanic White as the reference. The education variables were high school or similar, some college or similar, Bachelor's degree or higher, and none or elementary school as the reference. The income variables were $15,000–$29,999, $30,000–$44,999, $45,000–$59,999, $60,000–$74,999, $75,000+, missing, and <$15,000 as the reference.

We next included chemical concentrations measured inside the home of each participant. The model was

(3) $$\mathrm{logit}\left(p_{i}\right)=\beta_{0}+\beta_{1}\left(\sum_{j=1}^{C_{1}}w_{j1}q_{ij1}\right)+\sum_{k=1}^{K}\beta_{k}\left(\sum_{j=1}^{C_{k}}w_{jk}q_{ijk}\right)+z_{i}^{T}\phi ,$$

where there were K=7 groups of chemical exposures defined above in the Bayesian group index model, with each having health effect $\;\beta_{k}$. This extends the Bayesian grouped index model to include a neighborhood deprivation index along with multiple household-level chemical exposure groups, and allows for the development of different complex indices measured at different data scales (e.g., SES index at the neighborhood spatial scale and seven chemical indices at the household scale). This approach will reveal the relative importance of neighborhood deprivation compared with indoor chemical exposure effects.

To consider the spatial risk of childhood leukemia, we added *n* individual-level spatial random effects and exchangeable random effects to Equation (2) for unexplained spatial risk beyond what the neighborhood deprivation explains. The model was

(4) $$\mathrm{logit}\left(p_{i}\right)=\beta_{0}+\beta_{1}\left(\sum_{j=1}^{C_{1}}w_{j1}q_{ij1}\right)+z_{i}^{T}\phi +u_{i}+v_{i},$$

where the individual-level random effects $u_{i}$ and $v_{i}$ are for spatially unstructured and spatially structured heterogeneity, respectively, and spatial random effects are based on where the *i*th subject resides at the time of study enrollment.

We also added the indoor chemical groups to this model to determine if chemical concentrations measured inside the home would explain any spatial areas of elevated risk from the more common spatial risk model in Equation (4). This model was

(5) $$\mathrm{logit}\left(p_{i}\right)=\beta_{0}+\beta_{1}\left(\sum_{j=1}^{C_{1}}w_{j1}q_{ij1}\right)+\sum_{k=1}^{K}\beta_{k}\left(\sum_{j=1}^{C_{k}}w_{jk}q_{ijk}\right)+z_{i}^{T}\phi +u_{i}+v_{i},$$

where the chemical groups were as defined above for the model in Equation (3). We also used univariate Firth regressions [27,28] to explore the differences in explanatory variable effects for cancer status inside an elevated risk area versus the variable effects for being inside the elevated risk area for the complete study sample. Firth penalized regression was developed to mitigate the small sample bias found in traditional maximum likelihood logistic regression.

The models were completed with prior distributions for the parameters. The assumption of spatial correlation in area random effects was implemented through an *n*-dimensional multivariate normal prior v~MVN(0,∑), where ∑ is an *n* × *n* positive definite matrix with elements defined as $\underset{ij}{\sum }=\exp\left(-\psi d_{ij}\right)$ based on distance $d_{ij}$ between residential locations for different subjects. The parameter ψ controls the rate of decline of correlation based on distance and had a prior of ψ=Uniform(0.1,1), where the unit for this parameter is a scaling factor that acts on distances. The unstructured random effects received a standard prior of $u_{m}\sim Normal\left(0,\;\tau_{u}\right)$ with precision $\tau_{u}=1/\sigma_{u}^{2}$ and $\sigma_{u}\sim Uniform\left(0,100\right)$. The index effect and covariate effect parameters also received noninformative normal priors, $\beta_{k}\sim Normal\left(0,\tau_{k}\right)$ with precision $\tau_{k}=1/\sigma_{k}^{2}$ and $\sigma_{k}\sim Uniform\left(0,100\right)$. For any given index, the weights $w_{1k},\ldots ,w_{C_{k}k}$ were assigned a Dirichlet prior with parameters $\alpha_{jk}=\left(\alpha_{1k},\ldots ,\alpha_{C_{k}k}\right)$. This choice of prior ensures that the weights $w_{jk}\in \left(0,1\right)$ and $\overset{C_{k}}{\underset{j=1}{\sum }}w_{jk}=1$ to aid in the interpretation of the index. Inference on health effects and the relative importance of exposure indices is carried out through joint posterior distribution.

The Markov chain Monte Carlo (MCMC) method was used for model parameter estimation, and convergence to the posterior was assessed through the Gelman–Rubin diagnostic statistic using two chains in the R computing environment. We identified areas as being significantly elevated in risk using exceedance probabilities, which estimate how frequently the spatial odds at the *i*th location, $\theta_{i}=\exp\left(v_{i}\right)$, exceeded the null value of 1. The estimate of this probability uses the posterior distribution of the spatial odds at the location $\left(\theta_{i,m+1},\ldots ,\theta_{i,m+G}\right)$, where m represents the burn-in and G represents the number of posterior samples after the burn-in, and is calculated as $\hat{q_{i,U}}=\frac{1}{G}\sum_{g=m+1}^{m+G}I\left(\theta_{i,g}>1\right)$. Random effects with an exceedance probability of $\hat{q_{i,U}}\geq 0.90$ were considered to be of significantly elevated risk.

### 2.4. Selection Bias Sensitivity Analysis

As has been described previously, not all eligible cases and controls participated in the CCLS [11,13,26]. It has been noted that the controls participated at a lower rate than did cases and were less likely to participate in lower SES areas [29,30]. Therefore, we conducted a simulation study to evaluate the prospect that selection bias could lead to an observed area of significantly elevated childhood leukemia risk. We assessed the persistence of any observed spatial cluster by adding simulated participants to the set of study participants, in order to reach the full participation of eligible cases and controls. As there were 324 eligible cases and 277 participating cases with valid chemical measurements, we added $n_{case}=324-277=47$ simulated cases. Similarly, there were 407 eligible controls and 306 participating controls with valid measurements; therefore, we added $n_{control}=407-306=101$ simulated controls. We generated locations for the simulated participants using a two-step process. In the first step, we randomly sampled one census tract within the counties participating in the CCLS based on sampling weights we calculated to represent the population distribution. In the second step, we randomly sampled a location within the tract to represent a participant residence. For simulated cases, we weighted census tracts for sampling based only on population size, where the sampling weight was the population of the census tract divided by the total population of the census tracts within the study area. For simulated controls, in addition to population, we weighted census tracts according to two schemes that considered two SES variables previously used to represent SES [29]. In the first scheme, the tract weight was the product of its deciles for population, income deprivation (measured by inverse median household income [max(income)-income]), and education deprivation (measured by the percent of residents without a college degree). Specifically, the sampling weight for the ith tract was $w_{i}=\frac{inc_{i}\times edu_{i}\times pop_{i}}{\sum_{j=1}^{J}inc_{j}\times edu_{j}\times pop_{j}}$, where $inc_{i}$ is the decile for income deprivation, $edu_{i}$ is the decile for education deprivation, $pop_{i}$ is the decile for population, and there are J census tracts. We obtained tract-level covariate information from the American Community Survey, using five-year estimates for the period 2005–2009. In the second scheme, we preferentially sampled tracts with greater socioeconomic deprivation by applying the constants (1,1.5,1.5) to the deciles for population, income deprivation, and education deprivation, respectively. Specifically, the sampling weight for the ith tract in this scheme was $w_{i}=\frac{\left(1.5\times inc_{i}\right)\times \left(1.5\times edu_{i}\right)\times \left(1\times pop_{i}\right)}{\sum_{j=1}^{J}\left(1.5\times inc_{j}\right)\times \left(1.5\times edu_{j}\right)\times \left(1\times pop_{j}\right)}$.

We created 100 simulated datasets using the two weighting schemes for controls, and then tested for the occurrence of a spatial cluster using the commonly used methods of the local spatial scan and a Bayesian hierarchical spatial regression model. We used these approaches without any covariates, NDI, or chemical concentrations because covariates and chemical concentrations were not available for simulated participants, and the local spatial scan does not consider any covariates or risk factors. This meant that we had to first use the two approaches with the observed data to detect an observed cluster as a reference for comparison with the simulated datasets.

The first method of the local spatial scan used the SaTScan software and the rsatscan package in R, which assumes a Bernoulli distribution for case membership. The local spatial scan evaluates the likelihood ratio comparing the rates of cases and controls inside and outside a series of circles that are centered at each participant location. The likelihood ratio is constructed assuming an equal disease risk inside and outside the circle, and the circle's range in radius. The test identifies as the most likely cluster the circle with the maximum likelihood ratio test statistic, and performs inference using this circle and many Monte Carlo randomizations of case and control labels, producing a *p*-value of the rank of the most likely cluster over all randomizations.

The second method was the Besag–York–Mollie (BYM) Bayesian spatial regression model using the OpenBUGS software and the R2OpenBUGS package in R. This model assumes that for outcome variable $Y_{i}$ taking values of 1 or 0 for cases and controls, $Y_{i}∼Bernoulli\left(p_{i}\right)$, where $log\left(\frac{p_{i}}{1-p_{i}}\right)=\beta_{0}+u_{i}+v_{i}$. We assigned a flat prior to the intercept $\beta_{0}$, a normal prior to the spatially unstructured random effect $u_{i}$ with mean 0 and precision $\tau_{u},$ where $\tau_{u}=\frac{1}{\sigma_{u}^{2}}$ and $\sigma_{u}∼Unif\left(0,10\right)$, and an intrinsic Gaussian conditional autoregressive (CAR) prior to the spatially structured random effect $v_{i}$ with precision parameter $\tau_{v}∼Gamma\left(0.001,0.001\right)$. For the CAR prior, we defined adjacency for the spatially structured random effects using Voronoi polygons defined by participant locations. We used two chains to fit the model, burning in 15,000 iterations and sampling another 20,000 iterations from the joint posterior distribution.

For each dataset, we first recorded whether the model identified a spatial cluster. For the spatial scan model, this was defined as a *p*-value below 0.05 of observing the distribution of cases and controls in a circular window, under the null hypothesis of no difference in risk inside and outside the circle. For the BYM model, this was defined as a posterior exceedance probability of greater than 90 percent for the spatially structured random effect, specifically if $\hat{q_{v_{i}}}=\frac{\sum_{g=m+1}^{m+G}I\left(v_{i}^{\left(g\right)}>0\right)}{G}>0.90$, where m iterations are burned in and G iterations are sampled after the burn-in. If a cluster existed, we calculated the size of its convex hull in square kilometers, determined if it overlapped with the observed cluster, and calculated the percentage of its overlap. For each of the approaches, we compared the spatial cluster findings from the simulated datasets to those from the same approach with the observed dataset.

To assess potential selection bias on the NDI effect, we fitted the crude NDI model in Equation (1) to the same 100 datasets generated from the first scheme. It was not possible to fit any of the adjusted NDI models due to a lack of individual-level covariates for generated subjects. Therefore, this sensitivity analysis assessed the crude NDI effect after considering adding participants to overcome selection bias.

## 3. Results

### 3.1. Neighborhood Deprivation

**Figure 1:**
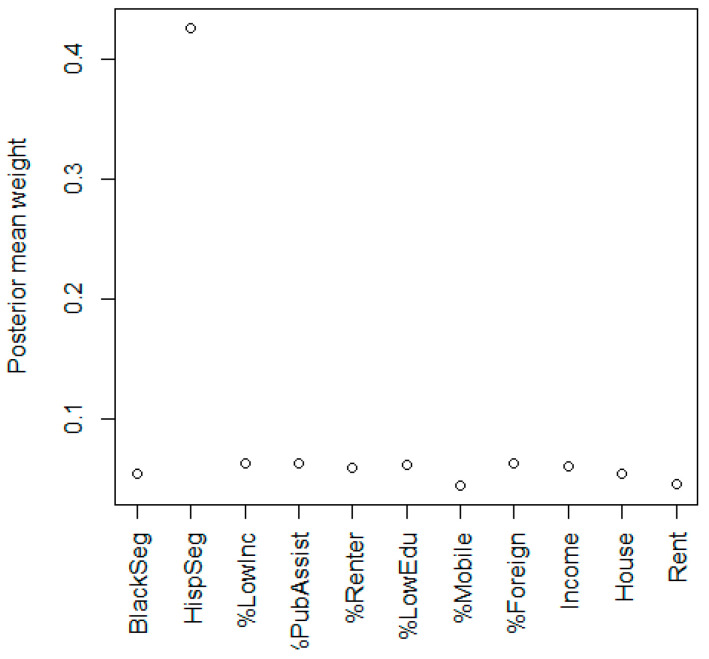
Posterior mean weights for variables in the crude model of neighborhood deprivation index for childhood leukemia. Note: components are Black segregation, Hispanic segregation, ratio of income to poverty <1, percent receiving public assistance income, percent of renters, percent with no high school diploma, percent in a different house than 1 year ago, percent foreign born, per capita income, median house value, and median gross rent.

**Figure 2:**
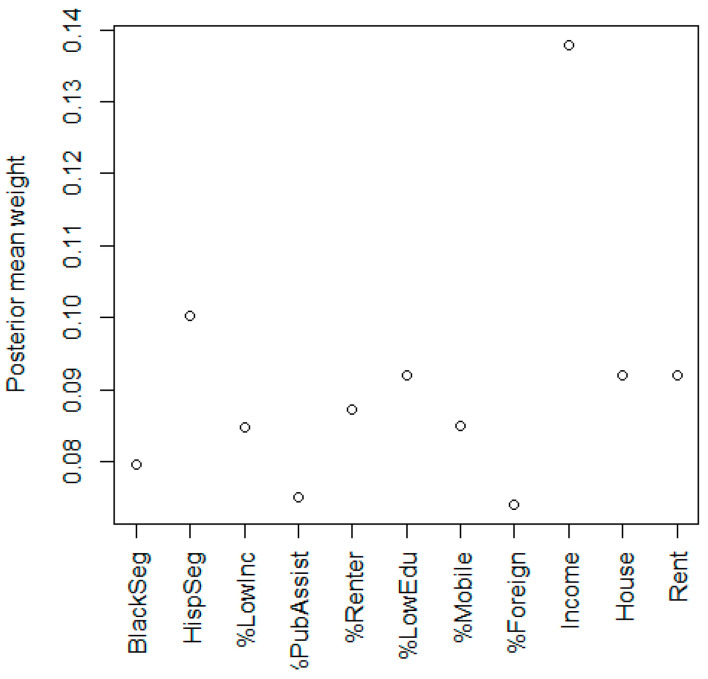
Posterior mean weights for variables in the covariate-adjusted model of neighborhood deprivation index for childhood leukemia. Note: components are Black segregation, Hispanic segregation, ratio of income to poverty <1, percent receiving public assistance income, percent of renters, percent with no high school diploma, percent in a different house than 1 year ago, percent foreign born, per capita income, median house value, and median gross rent.

In the crude model, there was a positive but not significant association with neighborhood deprivation and childhood leukemia, with an odds ratio (OR) of 1.06 and 95% credible interval (CI) of (0.98, 1.15) for a one-unit increase in the NDI. The NDI was dominated by Hispanic segregation with a weight of 0.43, while the other variables had much smaller weights (Figure 1). In the covariate-adjusted model (Table 1), the NDI OR was 0.97 with a 95% CI of (0.87, 1.07). After adjusting for covariates, the importance of Hispanic segregation decreased substantially to a weight of 0.10, and per capita income had the highest weight of 0.14 (Figure 2). The reduction in the Hispanic weight is explained by the adjustment of Hispanic ethnicity at the individual level. In the model that also adjusted for the groups of chemical exposures (Table 2), the NDI OR was 0.95 with a 95% CI of (0.85, 1.06). The adjusted models suggest an inverse association between NDI and childhood leukemia, but not a statistically significant one. Insecticides had a significant inverse association with an OR of 0.58 (0.35, 0.94).

### 3.2. Selection Bias Sensitivity Analysis for Neighborhood Deprivation

**Figure 3:**
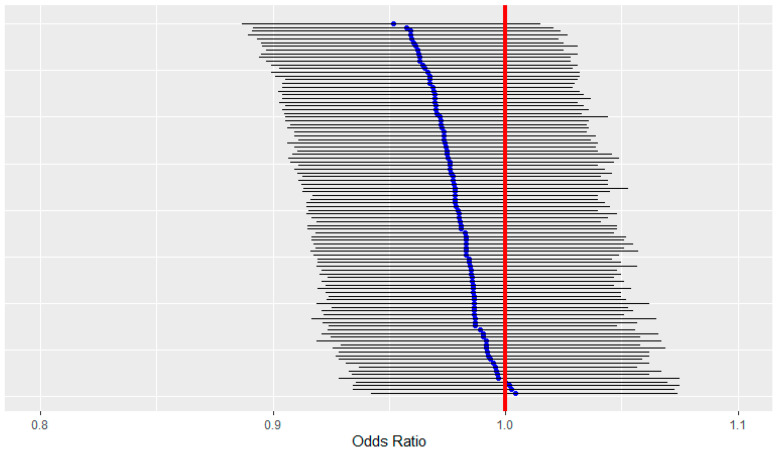
Neighborhood deprivation index odds ratio estimates in the blue circles and 95% credible intervals in black lines for each of the 100 simulated datasets from the first sampling scheme for participants.

**Figure 4:**
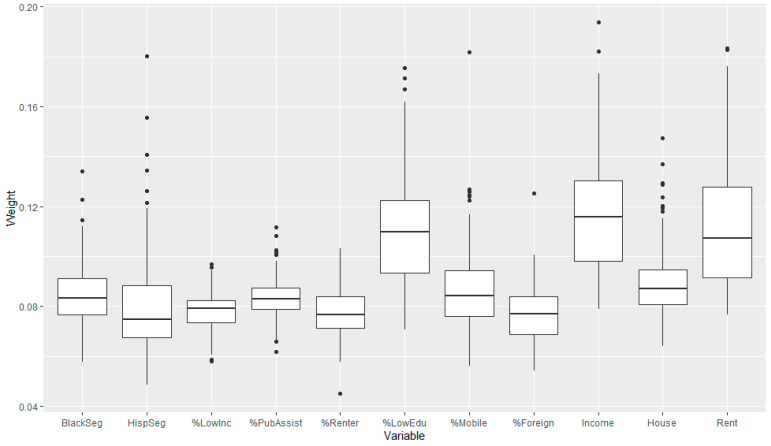
Distributions of neighborhood deprivation index component weights for the crude models for the 100 simulated datasets from the first sampling scheme for participants, where there are some large values above the body for most variables. Note: components are Black segregation, Hispanic segregation, percent of households with ratio of income to poverty <1 (below the poverty threshold), percent receiving public assistance income, percent of renters, percent with no high school diploma, percent in a different house than 1 year ago, percent foreign born, per capita income, median house value, and median gross rent.

Using the selected participants from the first sampling scheme, we found overall an inverse association of NDI and childhood leukemia in crude models over the 100 simulated datasets, with an average OR of 0.98 and 95% CI of (0.91, 1.05). The 95% credible intervals for each dataset always contained 1, while almost all OR estimates were less than 1 (Figure 3). The overall estimate was similar to that found in the adjusted NDI models for the observed CCLS dataset. The distribution of the NDI component weights from the 100 datasets show that per capita income had the highest weight overall (Figure 4), which is similar to the result for the covariate-adjusted NDI model for the observed data.

### 3.3. Spatial Risk

**Figure 5:**
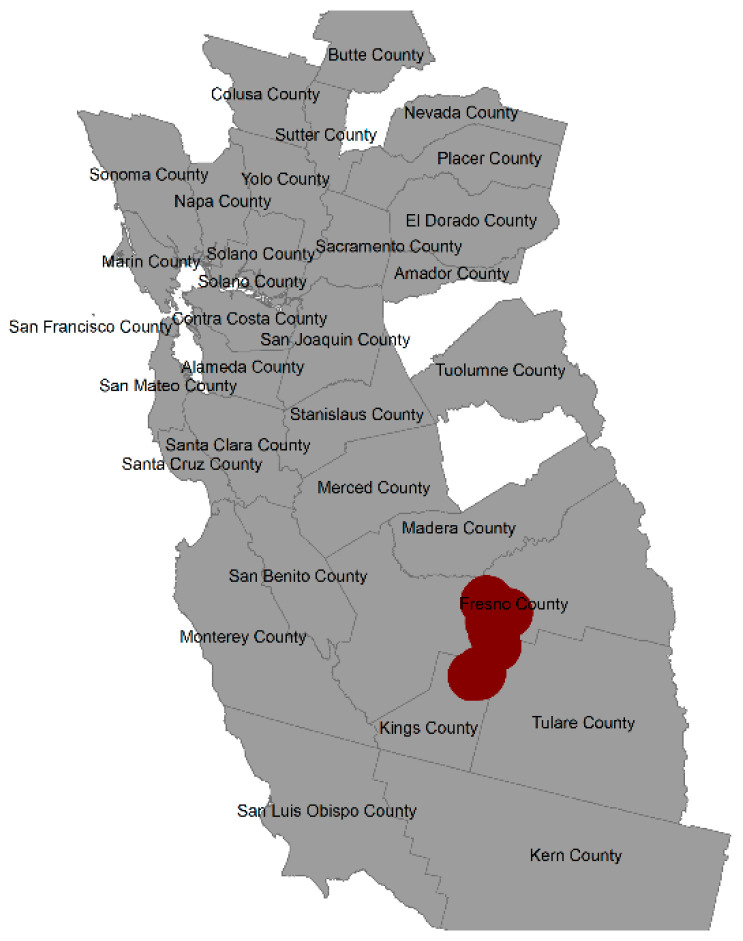
Area of elevated risk in red according to exceedance probabilities of spatial random effects in spatial risk model based on observed data.

The Bayesian index model with individual-level spatial random effects and exchangeable random effects (Equation (4)) found one area of elevated spatial risk based on 90% exceedance probabilities of the spatial random effects. There were 15 subjects located in the area of elevated risk, with 14 cases and one control. Of the 14 cases, 9 were Hispanic. The area of significantly elevated risk is illustrated in red in Fresno County and Kings County (Figure 5). The census block groups containing the 15 observations generally have high percentages of Hispanic population, with a mean of 66% and a range of 37% to 99% Hispanic. This elevated risk area was explained by adjusting for the chemical exposure groups (Equation (5)), as the exceedance probabilities of the spatial random effects for this area were no longer large. To explore this result, we examined the chemical group exposures for this area versus the entire study sample with univariate Firth regressions. We found much higher exposure coefficients for both insecticides and herbicides in the elevated exposure area (Supplemental Material Table S1) than in the entire study area (Supplemental Material Table S2), which could explain the relatively large number of cases in the area of elevated risk.

### 3.4. Selection Bias Sensitivity Analysis for Spatial Risk

**Figure 6:**
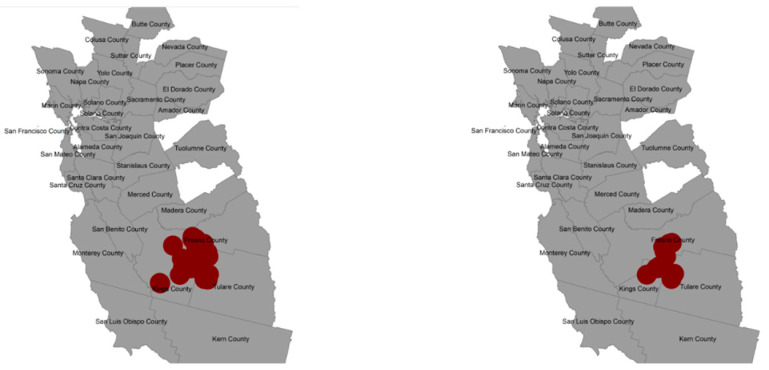
Significant spatial cluster in red detected using local spatial scan (**left**) and BYM model (**right**) based on the observed data.

**Figure 7:**
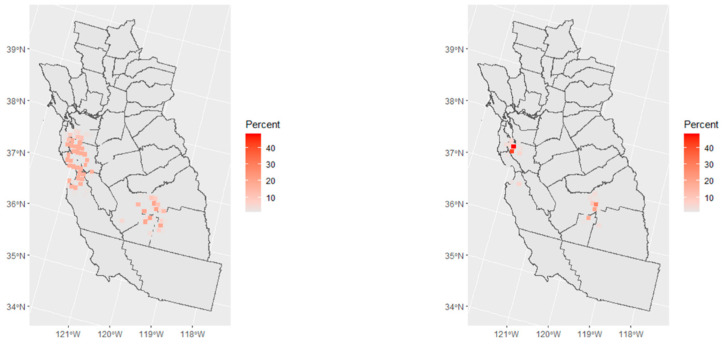
Percent of areas detected as significantly elevated risk over 100 simulated datasets from the first scheme using local spatial scan (**left**) and BYM model (**right**).

The two methods for cluster detection without covariates found a similar area as the model in Equation (4) for the observed data, albeit slightly larger and including Tulare County illustrated in red (Figure 6). However, the simulation study bias analysis results (Table 3) provided reduced evidence for the existence of the observed cluster when adding participants to achieve the full participation of eligible cases and controls. When using the local spatial scan statistic, a cluster was identified in 37 and 30 of the 100 datasets using the first and second weighting schemes for control participants, but only 20 and 12 of the 100 datasets produced a cluster that overlapped with the observed cluster from the same method. When using the BYM model, a spatial cluster was identified more frequently in 55 and 41 of the datasets using the first and second selection approach, but only 23 and 12 of the datasets had a cluster overlapping the observed one. As expected, both detection methods were less likely to find the observed cluster if controls had a higher probability of selection in lower SES areas. However, even in this participant selection approach the observed cluster was detected in 12 of the 100 datasets after overcoming selection bias, meaning that selection bias did not completely explain the observed spatial cluster. The maps of the number of times areas were detected as significantly elevated risk over the 100 datasets from the first scheme show two areas (Figure 7). One is the observed cluster area in Fresno and Kings Counties as well as Tulare County, depending on the method, and the other area is the south of San Francisco and overlapping with a spatial–temporal cluster that was previously detected in a California statewide analysis of childhood cancers including leukemia [31].

## 4. Discussion

In this study, we found that neighborhood deprivation was not positively associated with childhood leukemia after adjusting for individual level covariates in the observed data, and in sensitivity analysis for selection bias through sampling additional participants. We also found a significant spatial risk of childhood leukemia after adjusting for neighborhood deprivation and individual covariates in two counties in the observed data. However, the area of elevated risk was partly explained by selection bias in simulation studies that included more participating controls in geographic areas of lower SES. Interestingly, apart from selection bias, the area of elevated risk was explained when including groups of a large number of chemicals measured inside the home, and insecticides and herbicides had greater effects in the cluster area than overall.

In the CCLS, socioeconomic status was positively associated with likelihood of participation, and particularly so for controls [29]. This relationship is supported by a review of studies of childhood cancer that found that White control subjects were more likely to participate in interview-based case-control studies than non-White controls [32], as White participants tend to experience greater socioeconomic status than participants of other races [33]. In general, the finding of a spatial cluster that could be partly explained by selection bias has implications for spatial analyses that are subject to non-participation. Factors including SES, language differences, or lack of personal investment in a study, which may be more likely for eligible controls who may not have a personal connection to the disease under study, can drive non-response in eligible participants [29,34]. Interviews in the CCLS were conducted in English and Spanish per the eligibility criteria in the study, so speaking a language other than these two languages could increase the likelihood of non-participation. A recent review has suggested that this problem is more severe for controls than cases in studies of occupational risk factors [35]. In our study, non-participation of controls in a low-SES area contributed to identifying a spatial cluster of leukemia risk using several spatial statistical methods, whereas the addition of simulated controls led to not identifying the observed cluster in many datasets. In addition, preferentially sampling low-SES census tracts for controls led to less frequent identification of a cluster overlapping the observed one, compared to sampling controls with equal weights for population and SES factors. Thus, these findings suggest that spatial analyses should seek to evaluate the effect of selection bias on their results. This can occur by maximizing the likelihood of participation through offering financial incentives for participation, providing an accessible study experience that allows for participation in several languages and minimizes the time and effort required, or by assessing the effects of selection bias through adding simulated pseudo-participants in a manner similar to our own, or conducting registry-based analyses [31]. However, our approach of simulating participants does not allow for adjustment for important covariates or measured risk factors such as exposures within the home. Additionally, we note that it is possible to simulate participants on a variety of spatial scales, such as block groups.

Another factor not typically used in spatial analyses of cancer risk is exposure to a large number of chemicals inside the home. In our study, measured exposures to a large number of chemicals explained the observed spatial cluster that was not explained by neighborhood deprivation and individual covariates. While it is uncommon to measure so many exposures for individuals within the home, it explained the significant area of elevated risk even though the chemical groups did not have significant positive effects overall. However, it is more common in spatial analyses to identify patterns and significant unexplained risk areas that lead to follow-up studies to determine more individual-level exposures that could be related to the health outcome [36,37].

## 5. Conclusions

Our Bayesian analysis of childhood leukemia in the CCLS did not support a significant neighborhood deprivation effect in various models using observed and simulated data. In addition, an area of significantly elevated cancer risk in models of neighborhood deprivation and covariates was explained by higher effects of herbicides and insecticides among the seven chemical exposure groups inside the home. Selection bias was found to partially impact the detection of spatial areas of significant risk. The consideration of exposures and variables at different levels from multiple sources as well as potential selection bias are important for explaining observed spatial areas of elevated risk.

## Figures and Tables

**Table 1:** Odds ratios and 95% credible intervals for the covariate-adjusted neighborhood deprivation index (NDI) model for childhood leukemia.

Variable	Odds Ratio	2.5% CI	97.5% CI
NDI	0.97	0.87	1.07
Child’s age	1.00	0.91	1.11
Female	1.00	0.73	1.37
Child’s ethnicity			
Hispanic	1.30	0.86	2.08
Non-Hispanic	1.46	0.96	2.27
Household income			
$15,000–$29,999	1.04	0.53	2.06
$30,000–$44,999	0.90	0.46	1.70
$45,000–$59,999	0.87	0.42	1.70
$60,000–$74,999	0.52	0.22	1.10
$75,000 or more	**0.44**	**0.22**	**0.88**
Income missing	0.67	0.22	1.69
Mother’s education			
High school	1.12	0.62	2.29
Some college	1.09	0.59	2.20
Bachelor’s or higher	1.25	0.67	2.62
Mother’s age	1.01	0.98	1.04
Residence since birth	0.72	0.50	1.02

**Table 2:** Odds ratios and 95% credible intervals for the covariate-adjusted neighborhood deprivation index (NDI) and chemical exposure groups model for childhood leukemia.

Variable	Odds Ratio	2.5% CI	97.5% CI
PCBs	1.19	0.91	1.52
Insecticides	**0.58**	**0.35**	**0.94**
Herbicides	1.22	0.84	1.80
Metals	0.79	0.59	1.06
PAHs	1.21	0.98	1.52
Tobacco	0.84	0.67	1.04
PBDEs	1.25	0.82	1.86
NDI	0.95	0.85	1.07
Child’s age	1.01	0.91	1.11
Female	0.97	0.68	1.36
Child’s ethnicity			
Hispanic	1.32	0.83	2.18
Non-Hispanic	1.42	0.90	2.29
Household income			
$15,000–$29,999	1.02	0.46	2.20
$30,000–$44,999	0.73	0.32	1.50
$45,000–$59,999	0.74	0.31	1.60
$60,000–$74,999	0.41	0.15	1.01
$75,000 or more	**0.32**	**0.13**	**0.70**
Income missing	0.51	0.15	1.50
Mother’s education			
High school	1.33	0.64	3.14
Some college	1.31	0.62	3.22
Bachelor’s or higher	1.34	0.61	3.44
Mother’s age	1.01	0.98	1.05
Residence since birth	**0.68**	**0.46**	**1.00**

**Table 3:** Summary of spatial cluster detection results in simulations adding participants to the observed sample to evaluate selection bias. Notes: Weights refers to the sampling weights applied to census tracts for added control participants. Equal refers to equal weight given to income deprivation, education deprivation, and population for a census tract. SES refers to 50% greater weight given to income and education deprivation than to population for a census tract. Quantities in the table for Size and Overlap refer to the median and 95% percentile-based interval in square kilometers for the convex hull of the spatial cluster.

Method	Local Spatial Scan		Besag–York–Mollie	
Weights	Equal	SES	Equal	SES
% with Cluster	37.0	30.0	55.0	41.0
Size	0 (0, 5586)	0 (0, 4813)	0 (0, 7373)	0 (0, 8918)
Overlap	0 (0, 100.0)	0 (0, 92.0)	0 (0, 64.0)	0 (0, 30.0)
% with Overlap	20.0	12.0	23.0	12.0

## Data Availability

The CCLS data presented in this study are available on request from the senior author. The data are not publicly available due to privacy restrictions.

## Supplementary Materials

The following are available online at https://www.mdpi.com/article/10.3390/ijerph20043582/s1, Table S1: Univariate Firth regressions when the outcome was childhood leukemia among the subjects in the spatial area of elevated risk (n = 15); Table S2: Univariate Firth regressions when the outcome was childhood leukemia in the spatial area of elevated risk among all subjects in the analysis (n = 577).
