# Supplementary material for: Neighborhood Deprivation, Indoor Chemical Concentrations, and Spatial Risk for Childhood Leukemia
Source: Int J Environ Res Public Health. 2023 Feb 17;20(4):3582. doi: 10.3390/ijerph20043582 (PMC9968201; doi:10.3390/ijerph20043582)
Supplement: Supplementary file 1 [file ijerph-20-03582-s001.zip › ijerph-2168043-supplementary.pdf]

Supplemental Material

Neighborhood Deprivation, Indoor Chemical Concentrations, and Spatial Risk for Childhood Leukemia

David C. Wheeler [1,*], Joseph Boyle [1], Matt Carli [1], Mary H. Ward [2] and Catherine Metayer [3]

[1] Department of Biostatistics, School of Medicine, Virginia Commonwealth University, One Capitol Square, 830 East Main Street, Richmond, VA 23298-0032, USA

[2] Occupational and Environmental Epidemiology Branch, Division of Cancer Epidemiology and Genetics, National Cancer Institute, Rockville, MD 20850, USA

[3] School of Public Health, University of California Berkeley, Berkeley, CA 94704, USA

***** Correspondence: dcwheeler@vcu.edu; Tel.: +1-804-828-9827

Table S1. Univariate Firth regressions when the outcome was childhood leukemia among the subjects in the spatial area of elevated risk (N = 15)

Variable	Coefficient	95% CI	P-value
PCBs	0.23	(-2.33, 6.78)	0.8763
Insecticides	1.74	(-1.84, 6.61)	0.3468
Herbicides	2.92	(-1.53, 14.05)	0.2129
Metals	0.23	(-2.28, 4.49)	0.8698
PAHs	0.66	(-1.79, 4.10)	0.6179
Tobacco	-0.91	(-3.30, 1.05)	0.3302
PBDEs	2.06	(-1.71, 8.24)	0.2805
NDI Index	-0.08	(-1.92, 1.23)	0.9049
Child's Age	-0.29	(-2.02, 0.80)	0.5988
Female	0.55	(-2.56, 5.61)	0.7397
Child's Ethnicity: Hispanic	-0.55	(-5.61, 2.56)	0.7397
Non-Hispanic	-0.09	(-3.27, 4.98)	0.9586
Income: $15,000 - $29,999	-1.10	(-4.60, 4.05)	0.5756
$30,000 - $44,999	0.55	(-2.57, 5.61)	0.7397
$45,000 - $59,999	-1.65	(-6.70, 1.45)	0.3008
$60,000 - $74,999	NA (No subjects)		
$75,000 or more	NA (No subjects)		
Income Missing	NA (No subjects)		
Mother's education: High school	-0.51	(-3.77, 4.59)	0.7803
Some college	-1.65	(-6.70, 1.45)	0.3008
Bachelor's or higher	-0.09	(-3.27, 4.98)	0.9586
Mother's age	-0.01	(-0.21, 0.34)	0.9217
Residence since birth	-2.71	(-7.82, 0.48)	0.0952
Percent Hispanic	5.76	(-4.01, 31.77)	0.2660

Table S2. Univariate Firth regressions when the outcome was childhood leukemia in the spatial area of elevated risk among all subjects in the analysis (N = 577)

Variable	Coefficient	95% CI	P-value
**PCBs**	**-0.92**	**(-1.98, -0.14)**	**0.0188**
Insecticides	0.05	(-1.15, 1.23)	0.9355
Herbicides	0.45	(-0.58, 1.47)	0.3898
**Metals**	**-1.04**	**(-1.90, -0.23)**	**0.0113**
PAHs	0.07	(-0.53, 0.66)	0.8244
Tobacco	-0.50	(-1.24, 0.14)	0.1338
PBDEs	-0.03	(-1.06, 1.07)	0.9563
**NDI Index**	**0.59**	**(0.27, 0.98)**	**0.0002**
Child's Age	-0.06	(-0.37, 0.22)	0.6603
Female	-0.21	(-1.35, 0.82)	0.6912
**Child's Ethnicity: Hispanic**	**1.29**	**(0.25, 2.43)**	**0.0149**
Non-Hispanic	-0.02	(-1.40, 1.10)	0.9758
Income: $15,000 - $29,999	-0.10	(-2.32, 1.34)	0.9106
**$30,000 - $44,999**	**1.37**	**(0.22, 2.42)**	**0.0217**
**$45,000 - $59,999**	**1.63**	**(0.47, 2.69)**	**0.0075**
$60,000 - $74,999	-1.03	(-5.88, 1.01)	0.4034
**$75,000 or more**	**-3.25**	**(-8.10, -1.23)**	**0.0001**
Income Missing	-0.04	(-4.90, 2.04)	0.9792
Mother's education: High school	-0.43	(-2.07, 0.79)	0.5219
Some college	0.35	(-0.79, 1.39)	0.5275
Bachelor's or higher	-0.91	(-2.29 , 0.20)	0.1126
Mother's age	-0.02	(-0.11, 0.07)	0.6425
**Residence since birth**	**-1.64**	**(-3.28, -0.42)**	**0.0065**
**Percent Hispanic**	**5.02**	**(3.04, 7.38)**	**<0.0001**
